# REMOTION Blended Transdiagnostic Intervention for Symptom Reduction and Improvement of Emotion Regulation in an Outpatient Psychotherapeutic Setting: Protocol for a Pilot Randomized Controlled Trial

**DOI:** 10.2196/20936

**Published:** 2020-11-12

**Authors:** Laura Luisa Bielinski, Tobias Krieger, Franz Moggi, Leonie Trimpop, Ulrike Willutzki, Christoph Nissen, Thomas Berger

**Affiliations:** 1 Department of Clinical Psychology and Psychotherapy University of Bern Bern Switzerland; 2 University Hospital of Psychiatry and Psychotherapy University of Bern Bern Switzerland; 3 Department of Psychology and Psychotherapy Witten/Herdecke University Witten Germany

**Keywords:** blended therapy, internet-based intervention, emotion regulation, transdiagnostic, online therapy

## Abstract

**Background:**

Emotion regulation has been identified as an important transdiagnostic factor relevant to the treatment of mental health disorders. Many empirically validated psychotherapeutic treatments incorporate elements targeting emotion regulation. Most of these treatment approaches are conceptualized as standard face-to-face treatments not as blended treatments, which include an internet-based intervention.

**Objective:**

The aim of this study is to examine, for the first time, a new internet-based intervention—REMOTION—that will be provided transdiagnostically, as an add-on to psychotherapy, to provide a blended treatment format.

**Methods:**

A total of 70 participants will be assigned (1:1 allocation ratio) to either the intervention group (REMOTION + psychotherapy) or the treatment-as-usual group that receives psychotherapy alone. To maximize external validity, a typical outpatient treatment sample of patients diagnosed with a range of disorders such as depression, anxiety disorders, and adjustment disorder will be recruited from a university outpatient clinic. Patients with bipolar disorder, psychotic disorders, or acute suicidality will be excluded from the study. The feasibility and potential effectiveness of the intervention will be examined by assessing data at baseline, 6 weeks (post), and 12 weeks (follow-up). The primary outcome is general symptom severity, assessed with the Brief Symptom Inventory. Secondary outcomes are emotion regulation, depressive symptoms, anxiety symptoms, health related quality of life, well-being, and a variety of feasibility parameters. Quantitative data will be analyzed on an intention-to-treat basis.

**Results:**

Participant recruitment and data collection started in February 2020, and as of November 2020, are ongoing. Results for the study are expected in 2022.

**Conclusions:**

This pilot randomized controlled trial will inform future studies using transdiagnostic blended treatment.

**Trial Registration:**

ClinicalTrials.gov NCT04262726; http://clinicaltrials.gov/ct2/show/NCT04262726

**International Registered Report Identifier (IRRID):**

DERR1-10.2196/20936

## Introduction

### Emotion Regulation and Mental Health

According to Gross [1], emotion regulation refers to “the processes by which individuals influence which emotions they have, when they have them, and how they experience and express these emotions.” Emotion regulation can describe how individuals modulate the intensity or duration of an emotion and or the quality of an emotional response; it is often a conscious process, however, can also be an unconscious one [2].

According to several theoretical frameworks, successful emotion regulation is associated with positive health outcomes [3]. In recent years, research has continually shown that emotion regulation is an important transdiagnostic factor relevant to the treatment of mental health disorders [3-5]. Emotion regulation deficits play a role in the development, maintenance, and treatment of a variety of mental health disorders [6]. A number of empirically validated psychotherapeutic treatments incorporate elements of emotion regulation. For example, elements concerning emotion regulation can be found in the Unified Protocol for Transdiagnostic Treatment of Emotional Disorders [7], dialectical behavior therapy [8], accelerated experiential-dynamic psychotherapy [9], and emotion-focused therapy [10].

### Treatments Specifically for Emotion Regulation

Over the past years, there has also been an increase in treatment programs which explicitly target emotion regulation in order to improve mental illness symptoms. Many of these treatments have been carefully developed, and effectiveness has been shown in a number of trials. Examples are acceptance-based emotion regulation group therapy [11]; affect regulation training [12]; emotion regulation therapy [13]; Managing Emotions: Emotions Under Control [14]; Group Therapy for the Improvement of Emotion Regulation Skills [15]; and Gross model–based emotion regulation strategies training on anger reduction [16].

Notably, some of these treatment concepts have also made links to findings from research in basic affective science. Berking and colleagues [17], for example, have integrated findings from affective neuroscience in affect regulation training; they present 7 neural “vicious cycles” important to emotion regulation which are then complemented with 7 skills that are trained in sequence for adaptive emotion regulation. The effects of affect regulation training have been shown in several studies [17,18]. Gratz and Gunderson [11] developed emotion regulation group therapy by using a definition that draws on theoretical literature on emotion regulation during childhood and development, and places emphasis on the control of behavior while experiencing an emotion instead of control of the emotion [11]. Emotion regulation group therapy has shown effectiveness in several studies, and mechanisms of change have also been studied [11,19,20]. Emotion regulation therapy [21,22] also includes links to affective science by targeting motivational awareness, the development of regulatory capacities, and contextual learning [23] while making reference to theory by Gross [1]. In line with Gross’ differentiation of antecedent and response-focused strategies, emotion regulation therapy first teaches individuals adaptive response-focused strategies and then antecedent-focused strategies [24].

### The Extended Process Model of Emotion Regulation

In 2015, Gross presented a valuable extension to the process model of emotion regulation, named the extended process model of emotion regulation [2], elaborating that emotion regulation is an interaction of valuation systems and identifying emotion regulation stages: identification, selection, and implementation. The identification stage is concerned with whether to regulate emotion, the selection stage is concerned with what strategy should be used to regulate emotion, and the implementation stage is concerned with implementing a specific tactic suited to the situation [2]. Furthermore, Gross [2] also mentions the importance of flexibility in emotion regulation, described as matching strategy to circumstance.

A valuable contribution by Gross et al [25] described how elements from the extended process model of emotion regulation pertain to mental illness; maladaptive affect regulation can arise from identification, selection, implementation, and monitoring decisions and individuals can benefit from different treatment aspects and exercises depending on which stage of the model is affected. The intervention in this study, REMOTION, aims to use the stages of emotion regulation of the extended process model of emotion regulation [2] as a general framework for a highly structured transdiagnostic intervention. This intervention aims to foster the use of emotion regulation strategies, while also addressing potential difficulties encountered at each stage of regulation and focuses on training flexibility in emotion regulation strategy use.

### Over- and Underregulated Emotional States

In the field of psychotherapy research, the distinction between over- and underregulated emotional states and its relevance to psychotherapy have been made explicit [8,10,15,26]. According to Corcoran and colleagues [27], “one way to classify psychiatric disorders is to consider the degree to which emotions, reported within their syndromal presentation, are over or underregulated.” Greenberg [28] states that one guiding factor for integrative psychotherapy interventions may be the type of affect dysregulation involved (too little or too much emotion). Moreover, “whether clients are under- or overregulated and which emotions are to be regulated and how are important issues in any treatment [29]” . In accordance with this view, the same patient can experience both over- and underregulated states, and also exhibit patterns linked more closely to one or the other. Linehan [8,30] developed, in detail, distress tolerance skills relevant to overwhelming underregulated states.

On the other hand, more recently, radically open dialectical behavior therapy was developed for individuals with disorders characterized by overcontrol [26,31,32]. Emotional loneliness is seen as an important problem in disorders characterized by overcontrol [31]. The treatment is aimed at increasing flexible responding, prosocial signaling, openness, and emotional expressiveness of patients while reducing rigid inhibitory control [32]. Within radically open dialectical behavior therapy, specific skills for individuals with overcontrol problems have been developed [26]. REMOTION provides explicit strategies for both over- and underregulated states as described by Linehan [8] and Lynch [26], in a blended therapy format.

### Internet-Based Interventions

Over the last decades, the use of internet-based interventions has increased rapidly in the health sector and also in psychotherapeutic treatment. Internet-based interventions have become a popular and effective treatment format for the treatment of a variety of mental health disorders in various countries [33-39]; this has been shown mainly for internet-based cognitive behavioral therapy interventions but also for other treatment contents [40]. Such treatments often allow for more flexibility and convenience in use for the patient [41]. With regard to internet interventions focusing on emotion regulation, González-Robles and colleagues [42] have very recently published a study investigating the effect of an emotion-focused, guided internet treatment in specialized care; the internet intervention was superior to treatment as usual on measures of depression, anxiety, and health-related quality of life.

### Blended Treatment

The combination of internet interventions with conventional face-to-face therapy (blended treatment) is only in its early stages, and studies in a routine care setting are rare. Available studies on blended treatment in routine patient care show positive effects or positive trends for blended treatment [43-47]. For example, a study by Berger and colleagues [34] was able to show that a combination of psychotherapy and internet-based treatment was more effective than psychotherapy alone. Furthermore, Rizvi and colleagues [48], piloted an adjunct treatment for dialectical behavioral therapy called DBT Coach. DBT Coach was given to patients with borderline personality disorder and substance use for 10 to 14 days during outpatient treatment. A decrease of depressive symptoms and general distress was reported [48]. Moreover, Lukas and colleagues [49] piloted a blended therapy emotion regulation approach for individuals with elevated levels of alexithymia that showed positive effects on reducing alexithymia scores.

According to Erbe and colleagues [46], in comparison to purely internet-based treatments or purely face-to-face therapy, blended treatments could offer the following benefits: cost-effectiveness, increased effectiveness of treatment, improved transfer to everyday life, and ability to reach individuals for whom sole face-to-face or internet-based approaches are not suitable. A further benefit of providing interventions in a blended therapy format instead of solely internet-based is the argument that patient emotion regulation is assisted by the therapeutic relationship in session [10,50].

### Study Objectives

This study aims to pilot a blended treatment that uses an internet-based transdiagnostic program to improve emotion regulation (REMOTION) as an add-on to outpatient face-to-face psychotherapy. REMOTION aims to incorporate a variety of elements from effective treatment approaches into the emotion regulation framework provided by Gross [2], while also making explicit to patients specific strategies for over- and underregulated states [8,10,26]. The study aims to make use of the benefits of blended therapy format in order to convey emotion regulation skills to patients who are in psychotherapy. REMOTION aims to be a resource for both patient and therapist. This study aims to evaluate the feasibility and first effects of a transdiagnostic, blended treatment focused on emotion regulation in a mixed outpatient sample.

## Methods

### Study Design

**Figure 1:**
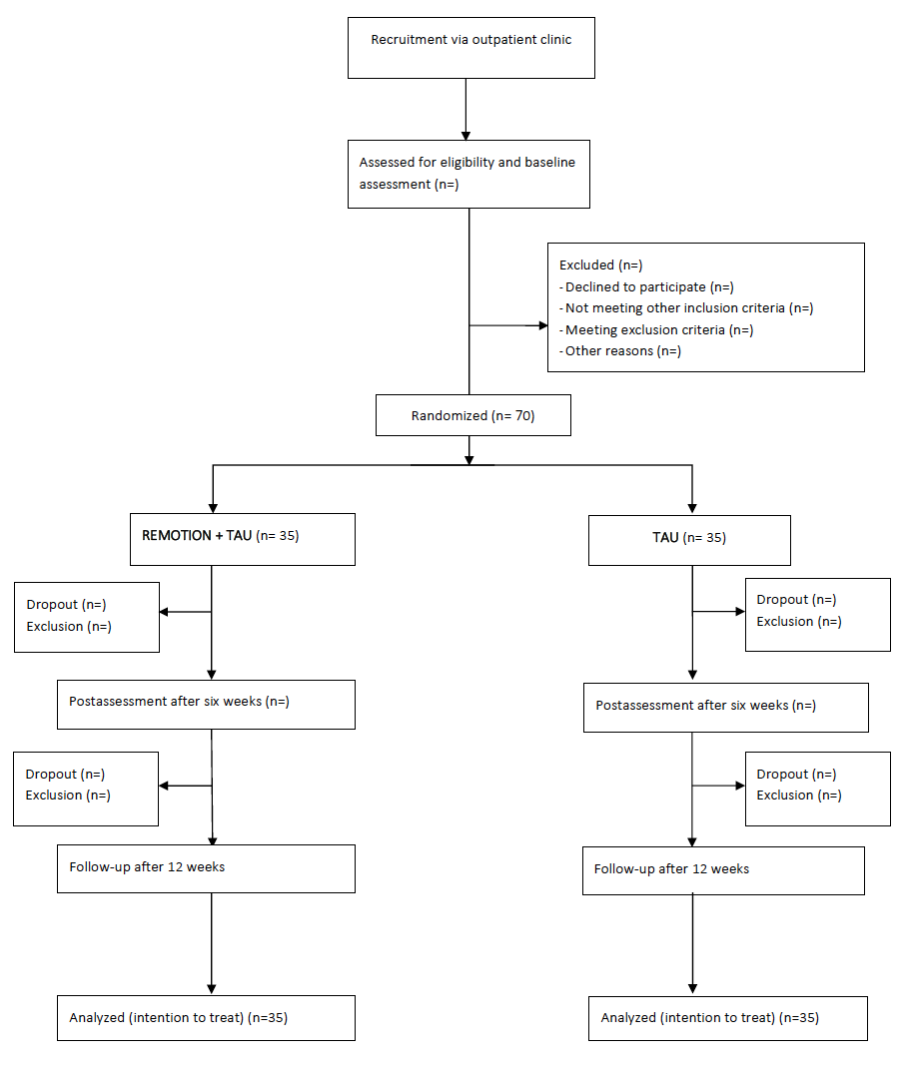
Participant flow. TAU: treatment as usual.

The study is a 2-arm pilot randomized controlled trial comparing an intervention group (REMOTION + psychotherapy) with a treatment as usual group (psychotherapy alone). Participants in the intervention group will immediately be given access to REMOTION whereas participants in the treatment as usual group will receive access to REMOTION after 12 weeks. Assessments will occur at baseline, 6 weeks (post), and 12 weeks (follow-up) for all participants. Assessment at 6 and 12 weeks will occur irrespective of whether the patient is still in face-to-face treatment. Figure 1 depicts the design of the trial. The single center trial will take place at the outpatient clinic of the Department of Clinical Psychology and Psychotherapy at the University of Bern, Switzerland.

### Sample Size

According to Sim and Lewis [51], 55 participants are the minimum necessary for a pilot trial. Moreover, according to Whitehead and colleagues [52], for a main trial designed with 90% power and 2-sided 5% significance, 25 participants per treatment arm are necessary in the pilot for standardized effect sizes that are small. Furthermore, previous unpublished data at the outpatient clinic for psychotherapy at the University of Bern has shown dropout rates of 15%. Therefore, a final sample size of 70 is planned for the study (35 per trial arm).

### Eligibility Criteria

The inclusion criteria are age over 18 years, in psychotherapeutic treatment, with mental illness, with internet access, and who provide written informed consent. The exclusion criteria are current participation in another intervention specifically for emotion regulation, a current episode or a history of psychotic disorders or bipolar disorder, acute suicidality, and not fluent in the German language.

### Recruitment, Randomization, and Blinding

Patients registering at the outpatient clinic will be informed about the study. Interested patients receive an information sheet, are invited to ask questions, and can provide written informed consent if they wish to participate. After study eligibility is proven, participants are randomly assigned to 1 of 2 groups (intervention or treatment as usual). Participants are randomized using a computerized random number generator and randomly permuted block sizes. The allocation schedule is generated by a researcher not involved in the research process and is unknown to the investigators and participants. There is no blinding implemented in the study, consistent with recommendations for the conduct of pragmatic randomized controlled trials in routine practice [53], in which the focus is on external validity and generalizability of the results to routine practice.

### Ethical Criteria and Ethics Committee

The study will be conducted according to local regulations and the Declaration of Helsinki. The study was approved by the ethics committee of the canton of Bern (ID 2019-01929). Written informed consent will be obtained from all patients. The trial is registered with clinicaltrials.gov (NCT04262726).

### Intervention

#### REMOTION

REMOTION is an internet-based program that was created at the University of Bern (by LLB in collaboration TB and with input from FM). A more detailed description of the program can be found in Table 1. It is a 6-part program (introduction and 5 modules), and the general sequence and components of the modules are based on the stages of emotion regulation in the extended process model [2]. A variety of elements from different evidence-based psychotherapeutic treatment approaches—dialectical behavior therapy [8], emotion-focused therapy [10], cognitive behavioral therapy [54], mindfulness based cognitive therapy [55], radically open dialectical behavior therapy [26], and Unified Protocol [7]—are incorporated into each module of REMOTION. Furthermore, the focus placed on overregulated as well as underregulated states as described by Greenberg [10] and Lynch [26].

**Table 1:** REMOTION content.

Module	Content
Introduction	Information about the structure of the intervention, about the theoretical background, and a user guide are provided in this module.
Psychoeducation	Information is provided about what emotions are, what their functions are, and what types of emotional experiences there are. The concept of emotion regulation is introduced, and the relationship between emotion regulation and mental illness is explored.
Identification	Emotional awareness, which is identified as key to the perception substep of the identification stage of emotion regulation [2], is explored in this module. If and when to regulate emotions, along with information on the value of emotion regulation, are introduced in this module.
Selection	This module shows patients what types of emotion regulation strategies are available. The focus is on the selection of an emotion regulation strategy [2]. The strategies—situation selection or modification, attentional deployment, change of cognitions, and response modulation [1]—are introduced in this module. Furthermore, strategies specific to over- and underregulated states [10,26,30] are also introduced.
Implementation	This module shows patients how the previously introduced strategies can be implemented, for example, translated to different tactics [2]. Exercises are introduced for every emotion regulation strategy, and advice is provided as to how these exercises can be implemented into daily life.
Monitoring/flexibility	Being able to modify strategies, being able to apply them flexibly, maintaining, switching, and stopping [2,25] are discussed in this module. Patients are encouraged to flexibly use strategies, to apply them to different contexts, to practice, and to try sequences or blends of strategies that work for them as individuals.

**Figure 2:**
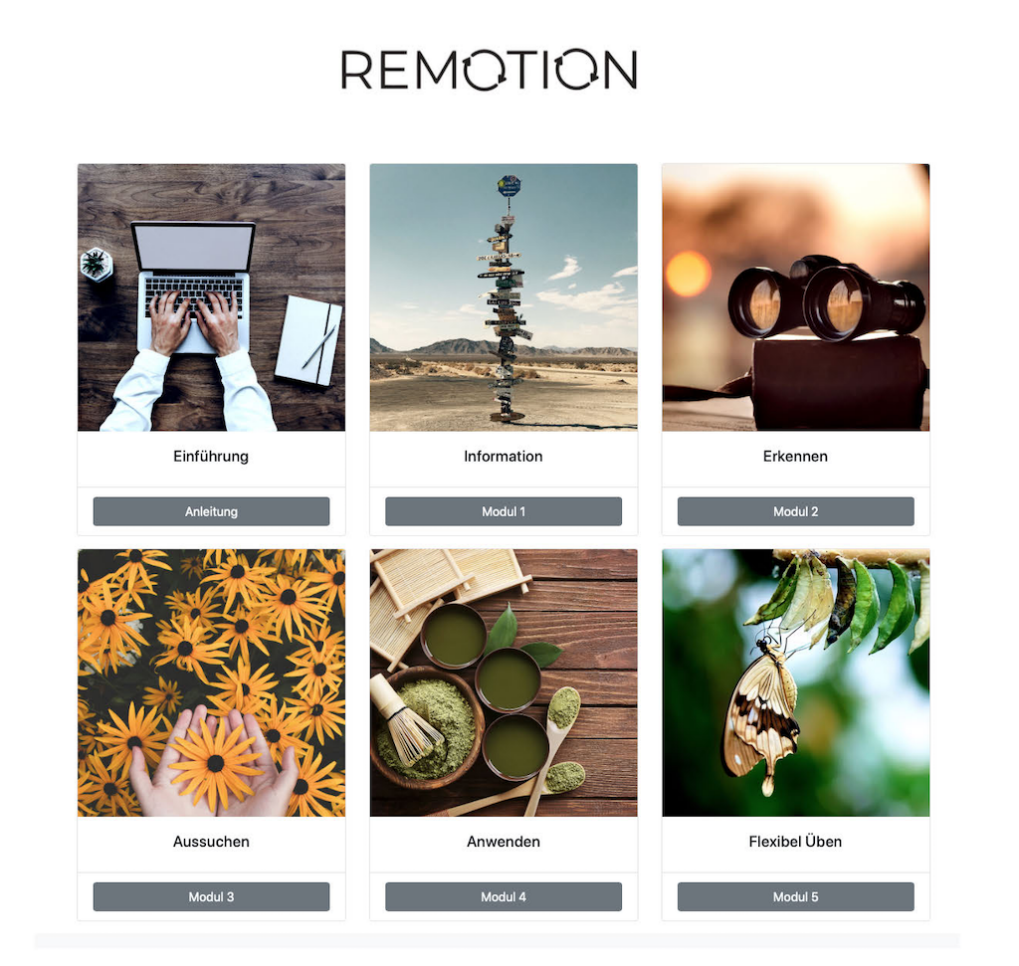
REMOTION homepage.

The program is provided to study participants on a platform (hosted by the University of Bern) free of charge and uses text, video, and audio material along with various exercises (Figure 2). Additionally, every week for 6 weeks, individuals in the intervention group receive an email reminding them which module they should be working on. After 9 weeks, patients will receive another email as a reminder to work on the program. Patients will also take part in their routine psychotherapy sessions while using REMOTION. Also, along with the internet-based program given to the patients, the therapists will receive information about the content of REMOTION in the form of an information booklet. The information given to the therapists is meant to explain the content of the internet-based program and provide suggestions as to how elements from the program can be integrated in face-to-face sessions. A detailed description of the information given to the therapist can be found in Table 2.

**Table 2:** Information provided to therapists.

Chapter	Content
Information about REMOTION	Information about the structure of REMOTION and theoretical background is provided for the therapists.
Information about each module	Each module and its content are outlined for the therapists. Therapists are informed about the exercises that patients complete in each module.
Information on using REMOTION exercises in face-to-face sessions	Therapists are provided with information as to how they can integrate specific exercises that patients have completed in the program, in their therapy sessions.

#### Treatment as Usual

Treatment as usual, in this study, consists of psychotherapy as administered in routine practice at the outpatient clinic of the Department of Clinical Psychology and Psychotherapy at the University of Bern. Psychotherapy at the outpatient clinic is an integrative form of cognitive behavioral therapy based on psychological therapy principles [56]. Individual case formulations are key in this treatment approach. This integrative form of cognitive behavioral therapy places a focus on empirically validated interventions and on the following general change factors in psychotherapy: clarification, resource activation, problem activation, and problem solving [57]. A further focus is placed on the analysis of problems and potentials for the therapeutic relationship, such as motive-oriented therapy relationship [58], and on plan analysis [59], which analyses the instrumental functions of patient behavior and experience. Patients in the treatment as usual group will not have access to REMOTION during the 12-week assessment period. Treatment sessions at the outpatient clinic usually take place once a week. The exact number of treatment sessions during the 12-week assessment period will be recorded for each patient.

### Therapists

Psychotherapy is administered by licensed psychotherapists who work at the outpatient clinic of the University of Bern and who have completed postgraduate training in psychotherapy at the University of Bern (Master of Advanced Studies in Psychotherapy), or by psychotherapists in said postgraduate training under regular supervision. All psychotherapists have master’s degrees in psychology. Psychotherapists in training have been in training for at least half a year and are regularly supervised. Therapists will be allocated to patients according to capacity at the outpatient clinic, a within-therapist design is used in the study.

### Measures

#### Overview

Items recording demographic information of patients will be recorded at baseline, post and follow-up. Also, as part of routine practice in the outpatient clinic, patient diagnostic status will be obtained during the study by conducting a Structured Clinical Interview I (German version) for Diagnostic and Statistical Manual of Mental Disorders (Fourth Edition) [60]. Furthermore, a qualitative interview with questions created specifically for the study will be conducted with a share of participants and therapists in the REMOTION group after 6 weeks. The purpose of this interview is to assess both experience and satisfaction with REMOTION, and thereby, should complement information from the questionnaires.

If deemed necessary, data collection will be aided by emails and phone calls in cases of poor data retention. A full description of all outcomes in the study is provided in the next sections. Unless stated otherwise, measures will be provided online.

#### Primary Outcome Measure

The primary outcome measure in this study is general symptom severity as measured with the Brief Symptom Inventory (German version) [61]. The Brief Symptom Inventory will be given to patients at baseline, post and follow-up. The Brief Symptom Inventory contains 53 items and is one of the most frequently used questionnaires to measure general symptom severity. A study [62] has shown that it has good psychometric properties, comparable with those of the Symptom Checklist-90-Revised instrument .

#### Secondary Outcome Measures

Emotion regulation will be assessed using 2 different instruments: (1) the German version [63] of Difficulties in Emotion Regulation Scale [64], a 36-item self-report questionnaire consisting of 6 subscales assessing difficulties in emotion regulation, and (2) Fragebogen zur standardisierten *Selbsteinschätzung emotionaler Kompetenzen* (SEK-27, *Emotion Competencies Questionnaire*) [65], a 27-item self-report instrument that addresses a range of emotion regulation skills, given to all patients in the study at baseline, 6 weeks, and 12 weeks. Moreover, therapists will also be asked to fill out ratings of patient emotion regulation. Good psychometric properties have been shown for the English [64] and German versions [63] of the Difficulties in Emotion Regulation Scale. The SEK-27 shows both good reliability and validity [65].

Depressive symptoms will be assessed with the German version [66] of the 9-item Patient Health Questionnaire, which is one of the most widely used self-report scales to assess depressive symptoms; criterion validity and change sensitivity have been reported [67].

Anxiety symptoms will be assessed with the German version [68] of the 7-item Generalized Anxiety Disorder Scale, a self-report measure that also shows good psychometric properties.

Health-related quality of life will be assessed with the German version [69] of the 12-item Short Form Health Survey, a frequently used, valid, reliable and change sensitive self-report questionnaire [69] used to assess both physical and psychological aspects of health-related quality of life.

Well-being will be assessed with the German version [70] of the World Health Organization Five Well-Being Index, a widely used economic instrument that shows excellent psychometric properties [70].

Feasibility parameters will be assessed in the study at different measurement timepoints: (1) The number of participants consenting to take part in the study and number of participants randomized will be recorded at the beginning of the study. (2) A previous study has shown that adherence may be an important factor in explaining the difference between effects of internet-based cognitive behavioral therapy in open recruitment and routine practice trials [71]; therefore, adherence to the program in this study will be assessed by number of modules completed at 6 weeks and 12 weeks for the REMOTION group, number of pages visited in the program at 6 weeks and 12 weeks for the REMOTION group, and number of exercises completed at 6 weeks and 12 weeks for the REMOTION group. (3) Usability of REMOTION will be assessed with the 10-item System Usability Scale [72] at 6 weeks and 12 weeks in the REMOTION group. (4) User experience of the REMOTION group will be recorded with the meCUE questionnaire [73], a self-report questionnaire that assesses user experience of products with 34 items at 6 and 12 weeks and with a qualitative interview that will be conducted with a share of the participants after the 6 week point, by telephone. (5) Patient attitudes toward online interventions will be assessed with the German version [74] of the Attitudes toward Psychological Online Interventions Questionnaire at baseline, 6 weeks, and 12 weeks for both study groups. (6) Satisfaction with the intervention will be assessed with the Client Satisfaction Questionnaire (in German [75] and adapted for internet interventions) at 6 and 12 weeks in the REMOTION group. Also, satisfaction will be assessed with qualitative interviews conducted with a share of the participants in the REMOTION group by telephone, after the 6-week point.

#### Other Measures

Therapeutic alliance measured with the German version [76] of the Working Alliance Inventory—short revised, a 12-item self-report scale that has shown good psychometric properties [76] will be given to all patients at 6 and 12 weeks. Patient self-compassion will be assessed with the German version [77] of the Self-Compassion Scale, a 26-item self-report scale that is both reliable and valid and will be given to all patients at baseline, 6 weeks, and 12 weeks. In order to assess negative effects of the intervention, an adapted version of the Inventory to Assess Negative Effects of Psychotherapy [78] for internet intervention will be used. Only 15 out of 21 items will be used; 6 items geared specifically at conventional psychotherapy will be exempt. The questionnaire is a self-report and will be given to REMOTION group patients at 6 and 12 weeks.

A variety of therapist variables will be recorded in the study, also including demographic data (experience, background, etc) and individual items on general use of emotion regulation interventions in therapy and general use of online interventions in therapy. In the REMOTION group, therapists perceived effect of REMOTION on therapy (attitude toward the intervention, use, satisfaction with the intervention, etc) will also be assessed with a set of items created specifically for the study. An interview will be conducted with a share of the therapists in the REMOTION group after the 6 week timepoint in order to further assess perceived effect of REMOTION on therapy. This interview will also collect data on therapist experience and satisfaction with REMOTION. It will be conducted per telephone.

Patient difficulties in emotion regulation and patient emotion compentencies will be rated by therapists using versions of the Difficulties in Emotion Regulation Scale (original [64], German version [63]) and SEK-27 [65], adapted specifically for this study, at the same measurement timepoints as patients. The wording of the questions is changed as little as possible from the original, but the questions are from an observer’s perspective about their patient.

Control of contamination between REMOTION and treatment as usual due to within-therapist design will be controlled in the following ways: the number of therapists who provide both REMOTION and treatment as usual therapies will be recorded, therapists who provide both conditions will be asked explicitly not to talk about REMOTION or use the REMOTION exercises provided in the REMOTION therapist booklet during treatment as usual therapy (a strategy utilized by studies in a review by Magill and colleagues [79]). Adherence to this condition will be recorded with items at post and follow-up for the therapists.

### Planned Analysis

Data will be analyzed on an intention-to-treat basis, meaning that all randomized patients will be included in the outcome analyses and missing data be handled accordingly. The primary outcome measure, general symptom severity, will initially be analyzed descriptively. Within- and between-group effect sizes will be calculated, and linear mixed models will be calculated. These models use all available data on a participant and estimate parameters of missing values. The various secondary outcomes will also be analyzed descriptively, then analyzed with linear mixed models, where applicable. With regard to feasibility parameters, the data will be characterized by descriptive statistics (means, standard deviations, and confidence intervals) in order to allow for comparison with other studies in the field. For categorical data, amount or percentage will be reported. The qualitative interviews generated for this study, will be analyzed using qualitative content analysis as recommended by Mayring [80]. Results will be reported in accordance with CONSORT (Consolidated Standards of Reporting Trials) [81] and CONSORT-EHEALTH [82].

## Results

The study was approved by the regional ethics committee in January 2020 and was registered with clinicaltrials.gov in February 2020. Participant recruitment and data collection started in February 2020, and as of November 2020, are ongoing. Results for the study are expected in 2022.

## Discussion

### General

This study aims to evaluate the benefit of adding a new transdiagnostic treatment tool, REMOTION, to outpatient psychotherapy. The study aims to provide a tool to improve emotion regulation transdiagnostically and to make use of the benefits of blended therapy as described, for example, by Erbe and colleagues [46]. More generally speaking, the results of this study could be used to improve transdiagnostic treatments of mental illness for patients and provide valuable information on the provision of blended therapy. Although many studies on internet-based and blended treatments are conducted in Switzerland, corresponding intervention formats are not implemented and available in routine practice. With regard to the concept of emotion regulation, to our knowledge, this is the first time an emotion regulation intervention structurally based on the stages of emotion regulation as specified in the extended process model of emotion regulation [2] is used with a clinical population in a blended psychotherapy setting. The application of a basic theoretical concept to a clinical psychotherapy context is a further strength of the study.

### Limitations

The following limitations of the study need to be considered. First, as this is a pilot trial, the number of patients examined in the study is small and thus only preliminary results on the effects of the treatment can be provided. However, this pilot trial can inform future larger studies that would be necessary to examine the efficacy or effectiveness in the future. Also, as this is a pilot study, no conclusions on specificity or mechanisms of change can be made. Moreover, most of the outcomes assessed in the study will be measured via self-report. In emotion regulation literature, the fact that self-report may be limiting has been described [64]. We have, as a result, tried to also include an observer rated assessment of emotion regulation by the therapist. Also, it should be considered, that unlike therapists for treatment as usual, therapists in the intervention group are encouraged to integrate elements of the REMOTION program into their psychotherapy sessions therapy. This may also lead to differences between the 2 study groups. Furthermore, it currently remains unclear what impact the COVID-19 pandemic may have on patient recruitment and data collection.

### Conclusions

REMOTION is a pilot randomized controlled study, assessing for the first time the feasibility and potential effectiveness of an internet-based emotion regulation treatment (REMOTION) as an add-on to psychotherapy in the form of a blended treatment. The study aims to make emotion regulation tools accessible to a broad range of patients and will provide insight into ways to improve psychotherapy for patients by the provision of internet-based tools. The strength of the approach lies in the application of the theoretical framework in a psychotherapy context and in the use of the treatment modality (blended). 

## Abbreviations

SEK-27: Fragebogen zur standardisierten Selbsteinschätzung emotionaler Kompetenzen (Emotion Competencies Questionnaire)
